# PLAU promotes fibroblast activation and keloid fibrogenesis through PLAUR–Dependent TGF–β/Smad signaling

**DOI:** 10.3389/fimmu.2026.1855430

**Published:** 2026-09-08

**Authors:** Qing An, Keai Li, Jingjing Gu, Huihui Pan, Jinru Song, Junyi Chen, Zhuohong Li, Yuanchao Li, Bin Yang

**Affiliations:** 1 Department of Dermatology and Rheumatology Immunology, Xinqiao Hospital of Army Medical University, Chongqing, China; 2 Dermatology Hospital, Southern Medical University, Guangzhou, China

**Keywords:** collagen expression, fibroblast, keloid, migration, PLAU, proliferation, TGF–β signaling

## Abstract

**Background:**

Keloids are fibroproliferative disorders conditions of the dermis with dysregulated immune responses contributing to disease progression. However, the causal inflammatory mediators and their pathogenic mechanisms remain undefined.

**Purpose:**

This study aims to identify causal inflammatory mediators for keloids and their pathogenic mechanisms.

**Methodology:**

Two–sample Mendelian randomization (MR) analyses were performed using genetic instruments for 91 inflammatory proteins as exposures and genome–wide association study (GWAS) summary statistics for keloid susceptibility as the outcome. Sensitivity analyses were conducted to assess the robustness of MR findings. Single–cell RNA sequencing (scRNA–seq) datasets from keloid tissues were analyzed to identify the cellular distribution of prioritized candidates. Functional validation was performed using immunohistochemistry (IHC), immunofluorescence staining, qRT–PCR, western blotting, siRNA–mediated knockdown, recombinant protein stimulation, and transcriptome sequencing. Pharmacological inhibition and rescue experiments were further performed to investigate the downstream signaling pathway.

**Results:**

MR analysis identified five inflammatory proteins with potential causal associations with keloid susceptibility, among which urokinase–type plasminogen activator (PLAU) was the only one consistently enriched in fibroblasts across two independent scRNA–seq datasets. PLAU expression was significantly elevated at both the mRNA and protein levels in keloid tissues and primary fibroblasts. PLAU knockdown in keloid fibroblasts reduced collagen I/III expression, proliferation, and migration, whereas recombinant PLAU stimulation enhanced these profibrotic phenotypes in normal dermal fibroblasts. Mechanistically, RNA–sequencing implicated activation of the TGF–β signaling pathway. Furthermore, inhibition of TGF–β receptor signaling attenuated PLAU–induced profibrotic responses, while exogenous TGF–β1 rescued the effects of PLAU depletion. Importantly, PLAUR knockdown attenuated PLAU–induced TGF–β/Smad activation and reduced PLAU–mediated enhancement of fibroblast activation.

**Conclusions:**

This integrative study identifies PLAU as a genetically supported and biologically relevant mediator of keloid fibrosis. PLAU promotes fibroblast activation through PLAUR–dependent regulation of the TGF–β/Smad signaling pathway, leading to enhanced collagen production, proliferation, and migration. These findings provide new insights into inflammatory regulation in keloid pathogenesis and highlight PLAU as a potential therapeutic target for keloid intervention.

## 1 Introduction

Keloids are pathological fibroproliferative scars characterized by excessive extracellular matrix (ECM) deposition and the ability to invade surrounding healthy tissue, causing substantial physical and psychological burdens for affected individuals. The pathogenesis of keloids is characterized by persistent fibroblast activation, including uncontrolled proliferation and excessive production of ECM components such as collagen I and collagen III (1, 2). Although chronic inflammation is recognized as a critical contributor to keloid development, the specific inflammatory mediators that are causally associated with keloid susceptibility and the mechanisms through which they regulate fibroblast activation remain incompletely understood (3–5). Recent advances in human genetics have provided new opportunities to identify disease–associated inflammatory factors and potential therapeutic targets (6).

Establishing causal relationships between inflammatory proteins and keloid susceptibility remains challenging in conventional observational studies because of confounding factors and reverse causation. Mendelian Randomization (MR) provides an analytical framework that uses genetic variants as instrumental variables (IVs) to infer potential causal relationships while minimizing these limitations (7, 8). Meanwhile, recent single–cell RNA sequencing (scRNA–seq) studies have revealed remarkable cellular heterogeneity within keloid tissues, identifying distinct profibrotic fibroblast subsets and altered immune microenvironments (9, 10). Integrating genetic causal inference from MR with cellular–resolution transcriptomic profiling by scRNA–seq provides a powerful strategy to prioritize inflammatory mediators involved in keloid pathogenesis and determine their potential cellular sources.

In this study, we performed two–sample MR analysis to investigate the potential contribution of 91 circulating inflammatory proteins to keloid susceptibility. Among the prioritized candidates, urokinase–type plasminogen activator (PLAU) emerged as a key candidate due to its genetic association with keloid susceptibility and consistent enrichment in fibroblast populations across independent keloid scRNA–seq datasets. Functional experiments demonstrated that PLAU regulates collagen expression, fibroblast proliferation, and migration. Furthermore, our findings suggest that PLAU promotes fibroblast activation through a PLAUR–dependent mechanism involving the TGF–β/Smad signaling pathway. Collectively, this study provides genetic and experimental evidence supporting PLAU as a potential mediator and therapeutic target in keloid fibrosis.

## 2 Materials and methods

### 2.1 Two–sample MR analysis

#### 2.1.1 Data sources

GWAS statistics for keloid (GWAS ID: ebi–a–GCST90018874) were obtained from a meta–analysis study including 668 cases and 481,244 controls of European ancestry (11). The GWAS data for 91 inflammatory proteins (GWAS ID: GCST90274758 – GCST90274848) were obtained from meta–analysis of 11 cohorts comprising 14,824 participants of European ancestry (6).

#### 2.1.2 Genetic IVs selection and two–sample MR analysis

Single–nucleotide polymorphisms (SNPs) used as IVs were selected based on the three key assumptions: relevance, independence, and exclusion restriction (7). SNPs associated with the exposure were collected at a genome–wide significance threshold (p < 1E–05). These SNPs were clumped by the clumping procedure of PLINK (version 1.90) with a linkage disequilibrium (LD) r^2^ threshold < 0.01 within 500 kb distance, and the LD r^2^ was calculated based on 1000 Genomes Projects as a reference panel (12). SNPs with F–statistics of < 10 and p–values (< 0.05) of MR Pleiotropy RESidualSum and Outlier (MR–PRESSO) method were removed due to weak IV strength and potential horizontal pleiotropy effects (13). Moreover, the reverse causality detection was done according to the descriptions above. All analyses were performed in R (version 4.1.3). Inverse variance weighting (IVW) was applied as the main method, and MR–Egger and weighted median–based methods were performed through the “TwoSampleMR” package. Cochran’s Q statistic was utilized among the selected IVs, and a p–value < 0.1 was considered to indicate heterogeneity. If the null hypothesis was rejected, the fixed–effect IVW was replaced by the random–effect IVW. A p–value < 0.05 was considered statistically significant. Scatter/funnel/forest plots and leave–one–out sensitivity analysis of MR analysis were utilized to show the results.

### 2.2 ScRNA–seq analysis of keloid

We utilized scRNA–seq data comprising keloid scar tissues (N = 3) and normal scar tissues (N = 3) generated by Deng Chengcheng et al. (GSE163973) (10), alongside scRNA-seq data encompassing four keloid scar tissues and their corresponding normal perilesional tissues produced by Liu Xu et al. (HRA000425) (9), to explore the differential expression levels of the selected proteins in keloids. The raw counts matrix was imported into R (version 4.3.1), and standard Seurat (version 4.3.0.1) protocols were used for downstream quality control and normalization. Cells with fewer than 200 or more than 6,000 detected genes, mitochondrial gene content greater than 15%, or red blood cell gene content greater than 3% were excluded. Then, the RunHarmony function from the Harmony package was applied to remove potential batch effects among samples processed in different batches. Principal Component (PC) Analysis was conducted, and the top 20 principal components on the 2,000 highly variable genes were used for dimensionality reduction and clustering (resolution = 1). Cells were clustered and visualized using uniform manifold approximation and projection (UMAP), and cell clusters were manually annotated according to expression of well–established cellular markers (9, 10, 14).

### 2.3 Experimental materials and methods

#### 2.3.1 Specimen collection

This study was approved by the Medical and Ethics Committees of Dermatology Hospital, Southern Medical University, and written informed consent to participate in our studies and for publication of their clinical characteristics was obtained from all participants. Tissue samples were collected during surgical procedures from patients who had a definite diagnosis and had not received medical or physical treatment before surgical treatment (details are shown in **Supplementary Table 1**).

#### 2.3.2 Real–time quantitative PCR (qRT–PCR)

The primer pairs for glyceraldehyde 3–phosphate dehydrogenase (GAPDH) and PLAU were designed through the Primer–BLAST part of NCBI (**Supplementary Table 2**). Total tissue or cell RNA was extracted using RNAiso Plus (Takara, Tokyo, Japan), and qRT–PCR was performed using SYBR Green Master Mix (Takara) to calculate the quantification cycle (Cq) value and normalize the relative mRNA expression in accordance with the Cq value of GAPDH.

#### 2.3.3 Immunohistochemistry (IHC) and immunofluorescence double–staining

All tissues were embedded in paraffin and sectioned at a thickness of 4.5 μm, and IHC staining for keloid and normal skin tissues were performed according to the manufacturer’s protocols for PLAU (#17968–1–AP, RRID: AB_2165202, 1:50, Proteintech). The 20X and 40X images were obtained through NDP.view 2 (Hamamatsu, Japan) to further analyze, and the immunoreactive score (IRS) was employed to evaluate PLAU expression. For immunofluorescence double–staining, paraffin–embedded keloid tissue sections were processed similarly for deparaffinization, rehydration, and antigen retrieval. After blocking, sections were incubated overnight at 4 °C with primary antibodies against PLAU (17968–1–AP, Proteintech, USA) and Vimentin (ab8978, Abcam, USA). After washing, sections were incubated with species–specific secondary antibodies, including Goat Anti–Rabbit IgG H&L (Alexa Fluor^®^ 488, ab150077, Abcam, USA) and Donkey Anti–Mouse IgG H&L (Alexa Fluor^®^ 555, ab150110, Abcam, USA). Nuclei were counterstained with DAPI, and fluorescence images were acquired using a fluorescence microscope. Colocalization analysis of PLAU and Vimentin signals was performed using ImageJ 2.9.0.

#### 2.3.4 Western blot (WB)

Tissues and fibroblasts were lysed in RIPA buffer (containing protease/phosphatase inhibitors; Fdbio, China). Protein concentration was measured using a BCA kit (Fdbio, China), with sample concentrations equalized before loading. Proteins were separated by SDS–PAGE and transferred to 0.45 μm PVDF membranes. After blocking non–specific binding, membranes were incubated overnight at 4 °C with primary antibodies against PLAU (17968–1–AP, Proteintech, USA), COL1A1 (91144S, CST, USA), COL3A1 (ab184993, Abcam, UK), Smad2 (5339S, CST, USA), Smad3 (9523S, CST, USA), p–Smad2 (Ser465/Ser467) (18338S, CST, USA), p–Smad3 (Ser423/425) (9520S, CST, USA) and GAPDH (RM2002, Ray, China). After washing, membranes were incubated with HRP–conjugated secondary antibodies (Anti–Rabbit IgG, RM3002, Ray, China; Anti–mouse IgG, 7076S, CST, USA). Protein bands were visualized using an ECL kit (Yeasen, China) and imaged with a Bio–Rad gel documentation system. Band intensity was quantified in ImageJ 2.9.0, normalized to GAPDH.

#### 2.3.5 Isolation and culture of primary fibroblasts

Primary fibroblasts were isolated from human keloid and normal skin tissues under sterile conditions according to a previously described protocol (15). Briefly, tissue specimens were carefully processed to remove non–dermal components, minced into small fragments, and subjected to enzymatic digestion (Trypsin; Gibco, USA). The isolated fibroblasts were cultured in high–glucose Dulbecco’s modified Eagle medium (DMEM; Gibco, USA) supplemented with 10% fetal bovine serum (FBS; Gibco, USA) at 37 °C with 5% CO_2_. Culture medium was replaced every 48 h, and cells were passaged after reaching 80–90% confluence. Fibroblasts between passages 3 and 7 were used for subsequent experiments. All procedures followed strict sterile protocols to prevent contamination. Post–treatment, cells were harvested for functional assays and protein analysis.

#### 2.3.6 Fibroblast transfection and stimulation

Fibroblasts were cultured in serum–free medium for at least 12 h before transfection or stimulation. According to the manufacturer’s instructions, fibroblasts were transfected with small interfering RNAs (siRNAs) targeting PLAU or PLAUR, or with non–targeting control siRNA (Ribobio, China), using RNAiMAX transfection reagent (Invitrogen, USA). The siRNA sequences targeting PLAU were as follows (1): genOFFTM st–h–PLAU_001(Ribobio, China): GTCACACCAAGGAAGAGAA (2); genOFFTM st–h–PLAU_002(Ribobio, China): CCACAGATCTGATGCTCTT. The siRNA sequence targeting PLAUR was [PLAUR Human Pre–designed siRNA Set A–1(HY–RS10648, MCE, China) G.A.U.C.C.A.G.G.A.A.G.G.U.G.A.A.G.A.A.U.U 3’, U.U.C.U.U.C.A.C.C.U.U.C.C.U.G.G.A.U.C.U.U 3’]. After 48 h of siRNA transfection, fibroblasts were treated with recombinant human PLAU protein (HY–P71050, MCE, China) at concentrations of 0.2 ng/mL or 2 ng/mL, or recombinant human TGF–β1 protein (HZ–1011, Proteintech, USA) at 10 ng/mL, according to the experimental design. For PLAUR–dependent signaling experiments, fibroblasts transfected with PLAUR–targeting siRNA or non–targeting control siRNA were subsequently stimulated with recombinant PLAU protein at 2 ng/mL to evaluate the role of PLAUR in PLAU–induced fibroblast activation. In addition, for TGF-β receptor inhibition experiments, normal dermal fibroblasts were treated with SB431542 (10 μM; HY-10431, MCE, China) before recombinant PLAU stimulation. Cells were harvested for subsequent Western blotting, EdU proliferation assays, Transwell migration assays, or RNA extraction according to the experimental requirements.

#### 2.3.7 EdU(5–ethynyl–2’–deoxyuridine) cell proliferation assay

Based on the protocol of the manufacturer, fibroblasts were seeded in 24–well plates, and their proliferative competence was determined using the BeyoClick™ EdU Cell Proliferation Kit coupled to Alexa Fluor 555 (Beyotime, China). The EdU–positive cells were then observed through fluorescence microscopy (A1 HD25/A1R HD25, Nikon, Japan).

#### 2.3.8 Transwell migration assay

In the upper chamber of Transwell inserts (#3422, Corning, USA), fibroblasts were seeded in serum–free DMEM. The bottom chamber held DMEM supplemented with 20% FBS. After 24 hours, migratory fibroblasts were immobilized by using 4% paraformaldehyde, stained with 0.1% crystal violet (Servicebio, China) for 30 minutes, and then viewed under an inverted microscope (ECLIPSE Ts2, Nikon, Japan).

#### 2.3.9 RNA sequencing and analysis

Total RNA was extracted from cells using RNAiso PLUS (TaKaRa, Japan) and submitted to Majorbio (Shanghai, China) for RNA sequencing. The sequencing platform employed was the Illumina NovaSeq X Plus, utilizing PE150 paired–end sequencing methodology. Subsequent analysis was performed using the clean data obtained after quality control. Transcriptomic analysis of keloid–derived fibroblasts following PLAU knockdown and primary normal dermal fibroblasts following recombinant PLAU stimulation were performed independently because they involved different fibroblast sources and distinct PLAU perturbation strategies. No direct statistical integration of the two expression matrices was performed. Differential expression and enrichment analysis were conducted separately for each experimental comparison. The edgeR software was utilized to perform the differential expression analysis, and the genes with p–value < 0.05 and |log2FC| ≥ 1.5 were considered differentially expressed. Gene Ontology (GO) functional enrichment analysis and KEGG PATHWAY enrichment analysis were conducted to elucidate the differences between samples at the gene functional level, and p–values less than 0.05 were defined as significantly enriched pathways among the differentially expressed genes.

#### 2.3.10 Statistical methods

Every experimental process was carried out individually, with at least three biological repeats. GraphPad Prism, version 9.4.1, was used to conduct statistical tests. Data are formatted with mean ± standard error of the Mean (SEM). Unpaired t–tests, one–way ANOVA or two–way ANOVA were conducted to achieve group comparisons. Categorical variables are presented in the form of frequencies (percentages) and were compared using chi–square or Fisher’s exact test. A p–value < 0.05 indicated statistical significance (*p < 0.05, **p < 0.01, ***p < 0.001, ****p < 0.0001).

## 3 Results

### 3.1 PLAU shows genetic evidence of association with keloid susceptibility and is enriched in keloid tissues

**Figure 1:**
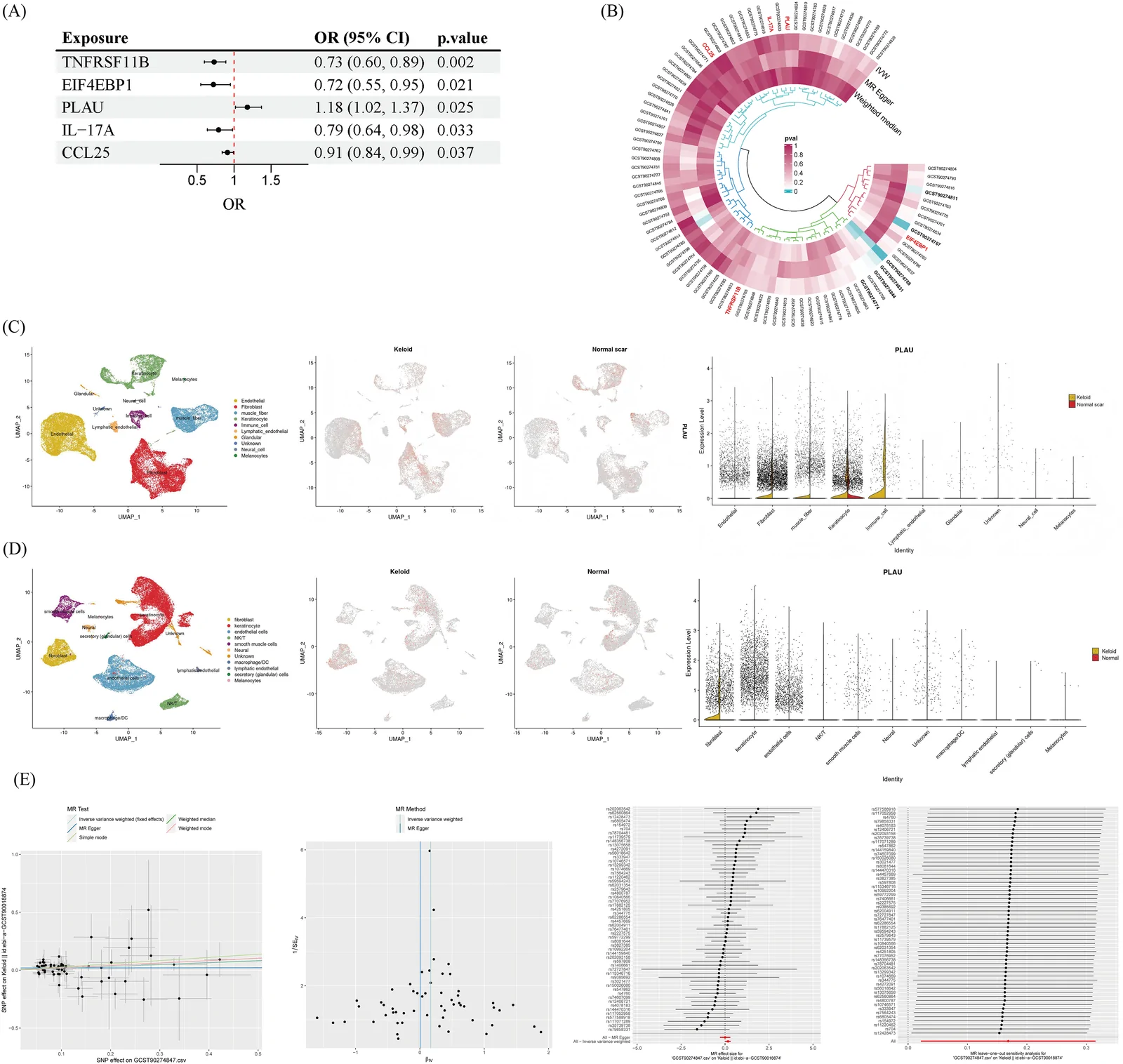
PLAU shows genetic evidence of association with keloid susceptibility and is enriched in keloid tissues. **(A)** Results of MR analysis between keloid and 91 inflammatory proteins. **(B)** Results of reverse MR analysis between keloid and 91 inflammatory proteins. **(C)** The UMAP plots and violin plots generated from the analysis of PLAU differential expression across various cell types in keloid versus normal scar tissue, based on single–cell RNA sequencing data published by Deng Chengcheng et al. **(D)** The UMAP plots and violin plots derived from the analysis of PLAU differential expression across distinct cell types between keloid and its adjacent normal skin, based on single–cell RNA sequencing data published by Liu Xu et al. **(E)** The scatter plot, funnel plot, forest plot and leave–one–out sensitivity analysis of MR analysis for PLAU and keloid. TNFRSF11B, Osteoprotegerin; EIF4EBP1, Eukaryotic translation initiation factor 4E–binding protein 1; PLAU, Urokinase–type plasminogen activator; IL–17A, Interleukin–17A; CCL25, C–C motif chemokine 25; IVW, inverse–variance weighted; MR, Mendelian Randomization; pval, p value; OR, Odds Ratio; UMAP, Uniform Manifold Approximation and Projection.

To identify inflammatory proteins potentially involved in keloid pathogenesis, we performed two–sample MR analysis using genetic instruments for 91 circulating inflammatory proteins and GWAS summary statistics for keloid susceptibility. The main results of the MR analysis are displayed in **Figure 1A** and **Supplementary Tables 3, 4**. Using IVW analysis as the primary method, genetically predicted higher levels of urokinase–type plasminogen activator (PLAU; OR: 1.18, 95%CI: 1.02–1.37, p = 0.025) were associated with increased susceptibility to keloid. However, higher osteoprotegerin (TNFRSF11B; OR: 0.73, 95% CI: 0.60–0.89, p = 0.002), eukaryotic translation initiation factor 4E–binding protein 1 (EIF4EBP1: OR = 0.72, 95% CI: 0.55 -0.95, p = 0.021), interleukin–17A (IL–17A; OR: 0.79, 95% CI: 0.64–0.98, p = 0.033), and C–C motif chemokine 25 (CCL25; OR: 0.91, 95% CI: 0.84–0.99, p = 0.037) levels were associated with lower odds for keloid.

As shown in **Figure 1B** and **Supplementary Table 5**, reverse–direction MR analysis did not identify evidence supporting reverse causation between keloid susceptibility and the five inflammatory proteins (TNFRSF11B, EIF4EBP1, PLAU, IL–17A, and CCL25).

To further prioritize candidate inflammatory mediators identified by MR analysis, we examined the expression patterns of their corresponding genes in two independent keloid scRNA–seq datasets. As illustrated in **Figures 1C, D**, PLAU was consistently enriched in fibroblast populations from both datasets compared with control tissues (p = 1.03E–05, p = 6.65E–60). In contrast, TNFRSF11B, EIF4EBP1, IL–17A, and CCL25 did not demonstrate consistent differential expression patterns in keloid tissues. Consequently, PLAU was selected for subsequent functional investigation.

We further conducted heterogeneity and sensitivity analysis on the MR results for PLAU and keloid. As shown in **Supplementary Table 3**, no significant heterogeneity was detected in the causal association between PLAU and keloid according to Cochran's Q (p = 0.949, I^2^ = 0). Furthermore, as displayed in **Figure 1E**, there was no directional pleiotropy for PLAU on keloid, as the intercept values were not significantly different from zero. To validate our findings, we analyzed causal associations using four other methods (MR Egger regression, Weighted–median, Simple mode and Weighted mode), and the trend directions displayed by the scatter plots strengthened the dependability of the causal relationship between PLAU and keloid. The funnel plot supported the results of the MR Egger intercept test and indicated that genetic pleiotropy did not bias causal effect estimates. We assessed the influence of each SNP on the analysis outcomes by using the leave–one–out method, and the overall error lines for PLAU on keloid did not change at all, implying that the causal impact of PLAU on keloid is very stable.

### 3.2 PLAU is upregulated in keloid tissues and predominantly localized in fibroblasts

**Figure 2:**
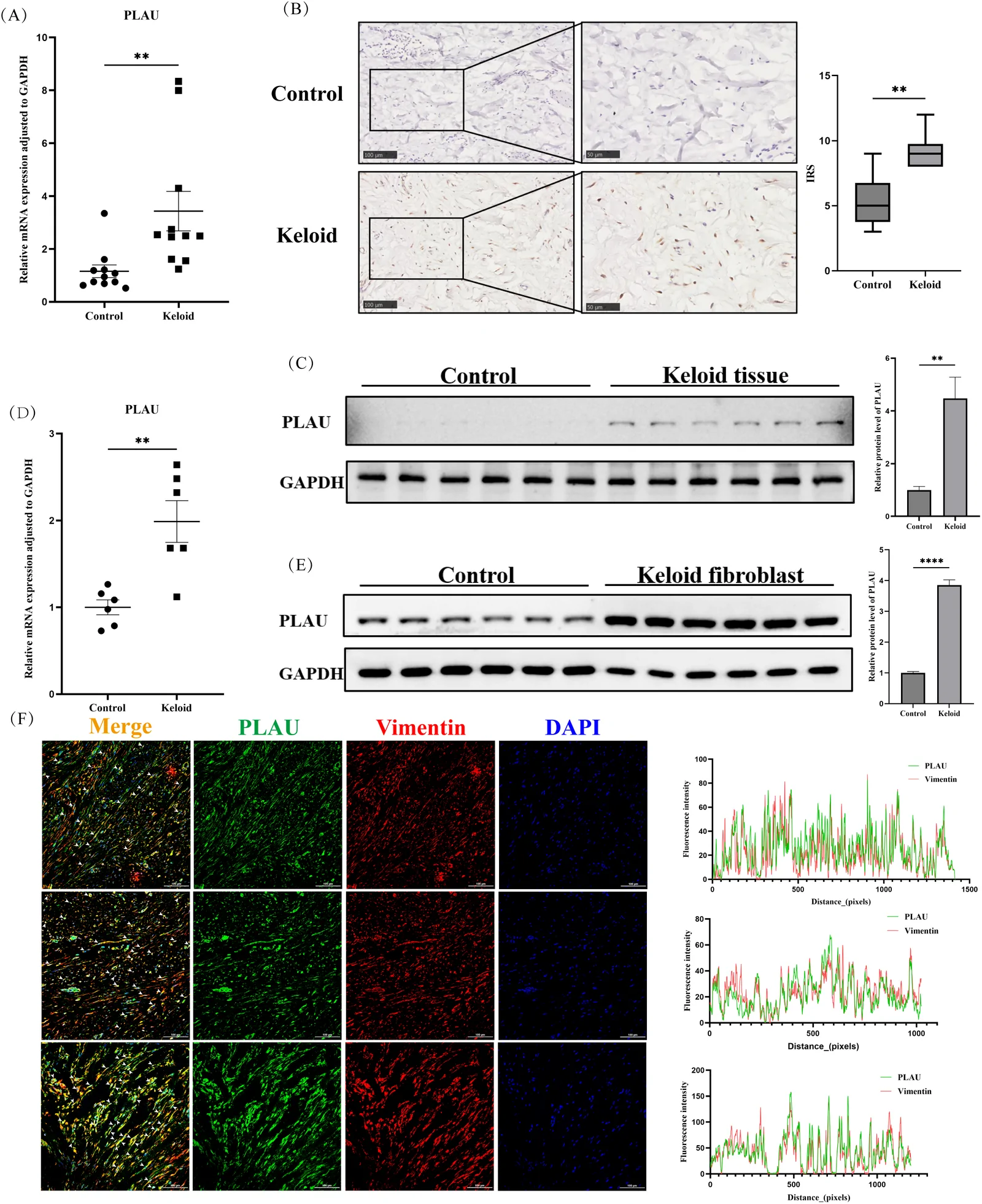
PLAU is upregulated in keloid tissues and predominantly localized in fibroblasts. **(A)** The mRNA levels of PLAU were assessed by qPCR in normal skin tissues and keloid tissues (N = 11 in each). **(B)** Immunohistochemical staining of PLAU in normal skin tissues and keloid tissues (N = 6 in each), Bar = 50 µm. **(C)** The protein levels of PLAU were assessed by western blot in normal skin tissues and keloid tissues (N = 6 in each). **(D)** The mRNA levels of PLAU were assessed by qPCR in NFs and KFs (N = 6 in each). **(E)** The protein levels of PLAU were assessed by western blot in NFs and KFs (N = 6 in each). **(F)** Representative immunofluorescence double staining showing PLAU (green) and Vimentin (red) expression in human keloid tissue sections (N = 3), nuclei were counterstained with DAPI (blue), merged images demonstrate substantial colocalization of PLAU and Vimentin signals (yellow), fluorescence intensity profiles along representative lines are shown to illustrate the spatial overlap between PLAU and Vimentin signals, Bar = 100 µm. **p<0.01, ****p<0.0001. PLAU, Urokinase–type plasminogen activator; GAPDH, Glyceraldehyde–3–phosphate dehydrogenase; NFs, Normal dermal fibroblasts; KFs, Keloid fibroblasts.

Based on the genetic evidence linking PLAU to keloid susceptibility identified by MR analysis, we next investigated PLAU expression at both the transcriptional and protein levels in keloid tissues and fibroblasts using qRT–PCR, IHC, and WB. Compared with normal skin tissues, keloid tissues exhibited significantly increased PLAU mRNA and protein levels (**Figures 2A–C**). Similarly, compared to normal dermal fibroblasts (NFs), upregulated mRNA and protein of PLAU were also detected in keloid fibroblasts (KFs) (**Figures 2D, E**). Thus, these findings show that PLAU is highly expressed in keloid compared with normal control subjects. Immunofluorescence double staining for PLAU and Vimentin confirmed the localization of PLAU in fibroblast populations (**Figure 2F**). Colocalization analysis from three independent keloid tissue sections demonstrated a strong PLAU–Vimentin correlation (Pearson r = 0.72) and substantial overlap (tM1 = 0.70), supporting fibroblasts as an important cellular source of PLAU in keloid tissues.

### 3.3 PLAU promotes fibroblast activation

**Figure 3:**
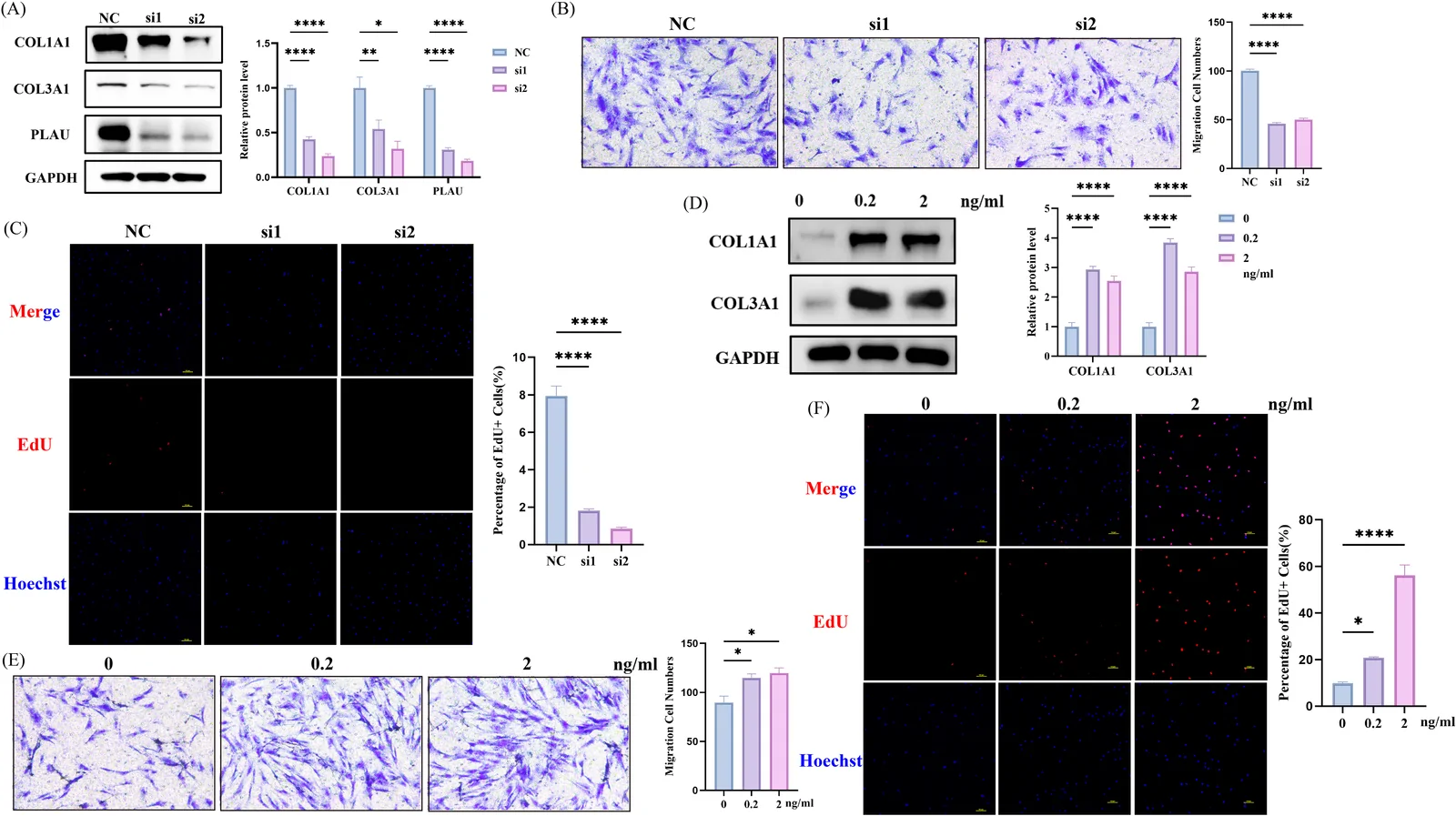
PLAU promotes fibroblast activation. **(A)** Western blot analysis showing the expression levels of collagen I and collagen III by KFs treated with PLAU–targeting siRNA or a non–targeting control. **(B)** Transwell assay shows the migratory ability of KFs treated with PLAU–targeting siRNA versus a non–targeting control. **(C)** EdU cell proliferation assay showing the proliferative capacity of KFs treated with PLAU–targeting siRNA or a non–targeting control. **(D)** Western blot analysis showing expression levels of collagen I and collagen III by NFs stimulated with recombinant PLAU protein at concentrations of 0.2 ng/mL and 2 ng/mL. **(E)** The Transwell assay shows the migratory ability of NFs stimulated with recombinant PLAU protein at concentrations of 0.2 ng/mL and 2 ng/mL. **(F)** EdU cell proliferation assay showing the proliferative capacity of NFs stimulated with recombinant PLAU protein at concentrations of 0.2 ng/mL and 2 ng/mL. *p<0.05, **p<0.01, ****p<0.0001, Bar = 100 µm. NC, non–targeting control; si, small interfering RNA (siRNA); PLAU, Urokinase–type plasminogen activator; COL1A1, Collagen type I alpha 1 chain; COL3A1, Collagen type III alpha 1 chain; GAPDH, Glyceraldehyde–3–phosphate dehydrogenase; EdU, 5–ethynyl–2’–deoxyuridine; NFs, Normal dermal fibroblasts; KFs, Keloid fibroblasts.

To investigate the functional effects of PLAU on fibroblast activation during keloid fibrosis, we performed PLAU knockdown in KFs and stimulated NFs with recombinant PLAU protein. As shown in **Figures 3A–C**, KFs subjected to PLAU–targeting siRNA knockdown exhibited decreased expression levels of collagen I and collagen III, as well as reduced cell migratory and proliferative capacities. Additionally, as shown in **Figures 3D–F**, stimulation with recombinant PLAU protein at concentrations of 0.2 ng/mL and 2 ng/mL upregulated the expression levels of collagen I and collagen III, along with enhanced cell migratory and proliferative capacities in NFs. To further determine the effects of endogenous PLAU depletion in NFs, we performed PLAU knockdown using two independent PLAU–targeting siRNAs. PLAU protein expression was reduced after siRNA treatment, with si2 producing a statistically significant reduction. In contrast to the pronounced changes observed in KFs, neither siRNA significantly altered COL1A1 or COL3A1 protein expression in normal dermal fibroblasts. Functional assays showed that si1 caused only modest, non–significant reductions in fibroblast proliferation and migration, whereas si2 significantly reduced both EdU incorporation and Transwell migration (**Supplementary Figure 1**). These findings indicate that endogenous PLAU also contributes to certain functional properties of NFs, although its influence on basal collagen I/III expression appears limited under the conditions examined.

### 3.4 Independent transcriptomic analyses implicate the TGF–β signaling pathway in PLAU–mediated fibroblast regulation

**Figure 4:**
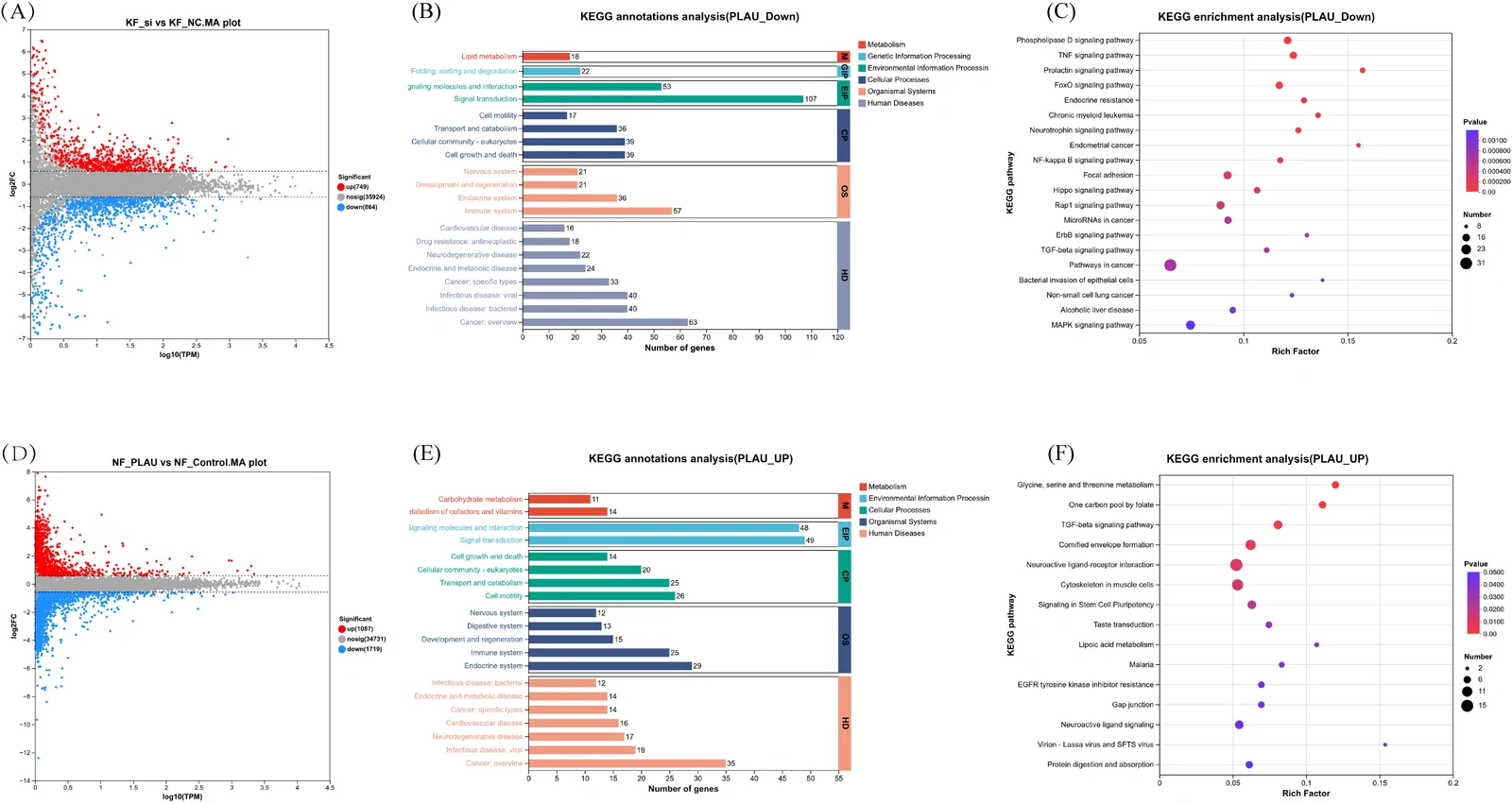
Independent transcriptomic analyses of PLAU perturbation in keloid and normal dermal fibroblasts. **(A–C)** Transcriptomic analysis of KFs following PLAU knockdown, **(A)** M–versus–A plot of differentially expressed genes following PLAU knockdown, **(B)** KEGG classification of genes downregulated following PLAU knockdown, **(C)** KEGG enrichment analysis of genes downregulated following PLAU knockdown. **(D–F)** Transcriptomic analysis of primary NFs following recombinant PLAU stimulation. **(D)** M–versus–A plot of differentially expressed genes following recombinant PLAU stimulation. **(E)** KEGG classification of genes upregulated following recombinant PLAU stimulation. **(F)** KEGG enrichment analysis of genes upregulated following recombinant PLAU stimulation. The TGF–β signaling pathway was independently identified in both experimental systems. PLAU, Urokinase–type plasminogen activator; KEGG, Kyoto Encyclopedia of Genes and Genomes; KFs, Keloid fibroblasts; NFs, Normal dermal fibroblasts.

To investigate the signaling pathways associated with PLAU–mediated fibroblast regulation, we performed two independent RNA–sequencing experiments. KFs subjected to PLAU knockdown and NFs stimulated with recombinant PLAU protein were analyzed separately because they involved different cellular backgrounds and distinct PLAU perturbation strategies. Pathway enrichment analysis of downregulated genes identified the TGF–β signaling pathway as a significantly enriched pathway in KFs with PLAU knockdown (**Figures 4A–C**). Conversely, recombinant PLAU stimulation in NFs induced transcriptional changes in which the TGF–β signaling pathway was also significantly enriched among upregulated genes (**Figures 4D–F**). Although these two datasets were not directly integrated statistically, their independent enrichment of TGF–β signaling following opposite PLAU perturbations provides convergent evidence supporting the involvement of TGF–β signaling downstream of PLAU.

### 3.5 TGF–β receptor inhibition attenuates PLAU–induced profibrotic responses

**Figure 5:**
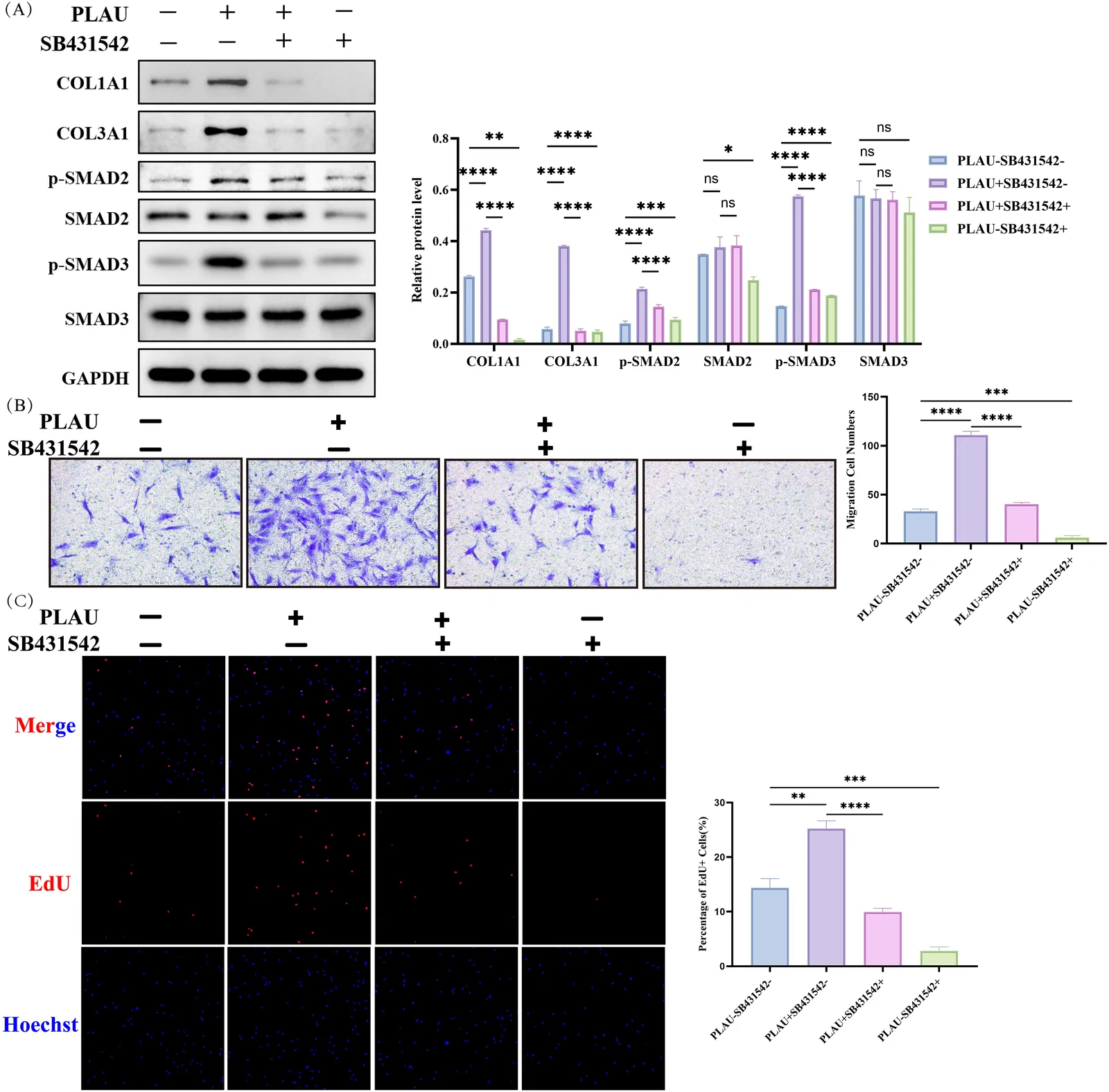
TGF–β receptor inhibition attenuates PLAU–induced profibrotic responses. NFs were stimulated with recombinant PLAU protein with or without the TGF–β receptor inhibitor SB431542. **(A)** Western blot analysis showing the expression levels of COL1A1, COL3A1, p–SMAD2/3 and SMAD2/3 under indicated conditions. **(B)** Representative images and quantitative analysis of Transwell migration assays showing the effects of SB431542 on PLAU–induced fibroblast migration. **(C)** Representative fluorescence images and quantitative analysis of EdU incorporation assays showing the effects of SB431542 on PLAU–induced fibroblast proliferation. These results demonstrate that inhibition of TGF–β receptor signaling attenuates PLAU–induced collagen expression, migration, and proliferation in fibroblasts. *p<0.05, **p<0.01, ***p<0.001, ****p<0.0001; ns: not significant. Bar = 100 µm. PLAU: Urokinase–type plasminogen activator; COL1A1: Collagen type I alpha 1 chain; COL3A1, Collagen type III alpha 1 chain; p, phosphorylated; SMAD, Suppressor of mothers against decapentaplegic; GAPDH, Glyceraldehyde–3–phosphate dehydrogenase; NFs, Normal dermal fibroblasts; EdU, 5–ethynyl–2’–deoxyuridine.

To experimentally validate the involvement of the TGF–β signaling pathway identified by transcriptomic analysis, NFs stimulated with recombinant PLAU protein were treated with the TGF–β receptor inhibitor SB431542. As shown in **Figures 5A–C**, inhibition of TGF–β receptor signaling significantly attenuated PLAU–induced increases in collagen I and collagen III expression, as well as the enhanced migratory and proliferative capacities of fibroblasts. These findings indicate that TGF–β signaling is required for PLAU–mediated profibrotic fibroblast activation.

### 3.6 Exogenous TGF–β1 restores fibroblast activation following PLAU knockdown

**Figure 6:**
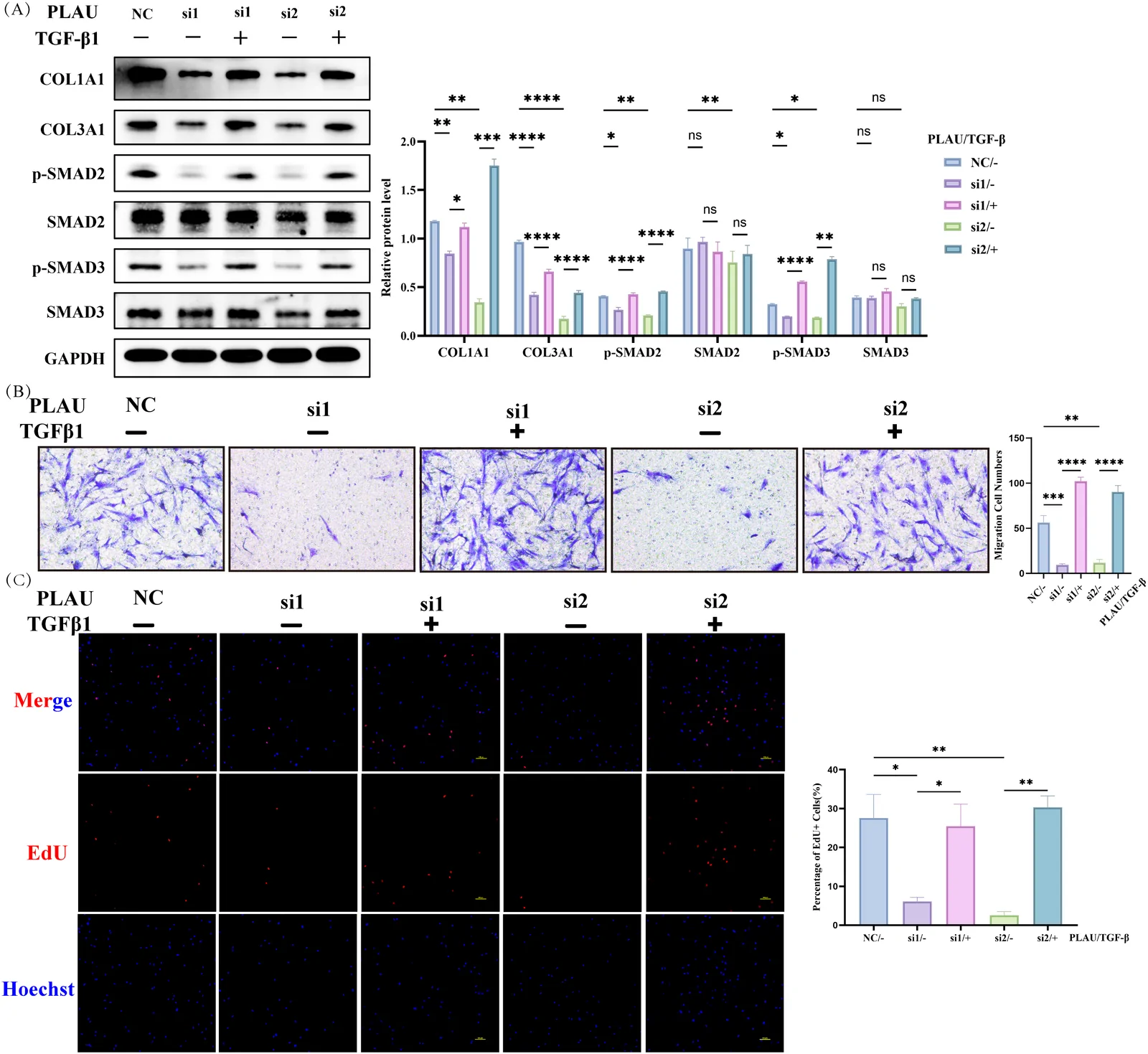
Exogenous TGF–β1 restores fibroblast activation following PLAU knockdown. KFs were transfected with PLAU–targeting siRNA or non–targeting control siRNA, followed by treatment with or without recombinant human TGF–β1 protein. **(A)** Western blot analysis showing the expression levels of COL1A1, COL3A1, p–SMAD2/3 and SMAD2/3 under the indicated conditions, quantification of protein expression is shown on the right. **(B)** Representative images and quantitative analysis of Transwell migration assays evaluating fibroblast migratory capacity after PLAU knockdown with or without TGF–β1 stimulation. **(C)** Representative fluorescence images and quantitative analysis of EdU incorporation assays evaluating fibroblast proliferative capacity after PLAU knockdown with or without TGF–β1 stimulation. These results demonstrate that exogenous TGF–β1 partially restores the impaired profibrotic responses caused by PLAU depletion, supporting TGF–β signaling as a downstream mediator of PLAU–induced fibroblast activation. *p<0.05, **p<0.01, ***p<0.001, ****p<0.0001; ns, not significant. Bar = 100 µm. PLAU, Urokinase–type plasminogen activator; TGF–β1, Transforming growth factor beta 1; NC, non–targeting control; si, small interfering RNA (siRNA); COL1A1, Collagen type I alpha 1 chain; COL3A1, Collagen type III alpha 1 chain; p, phosphorylated; SMAD, Suppressor of mothers against decapentaplegic; GAPDH, Glyceraldehyde–3–phosphate dehydrogenase; KFs, Keloid fibroblasts; EdU, 5–ethynyl–2’–deoxyuridine.

To further investigate whether TGF–β signaling acts downstream of PLAU, recombinant TGF–β1 protein was added to KFs following PLAU knockdown using PLAU–targeting siRNA. As shown in **Figures 6A–C**, exogenous TGF–β1 partially restored the reduced collagen I and collagen III expression caused by PLAU depletion. Moreover, TGF–β1 treatment rescued the impaired migratory and proliferative capacities of fibroblasts following PLAU knockdown. These findings further support that TGF–β signaling functions as a critical downstream pathway mediating PLAU–induced fibroblast activation.

### 3.7 PLAU–induced TGF–β/Smad activation and fibroblast profibrotic responses are PLAUR–dependent

**Figure 7:**
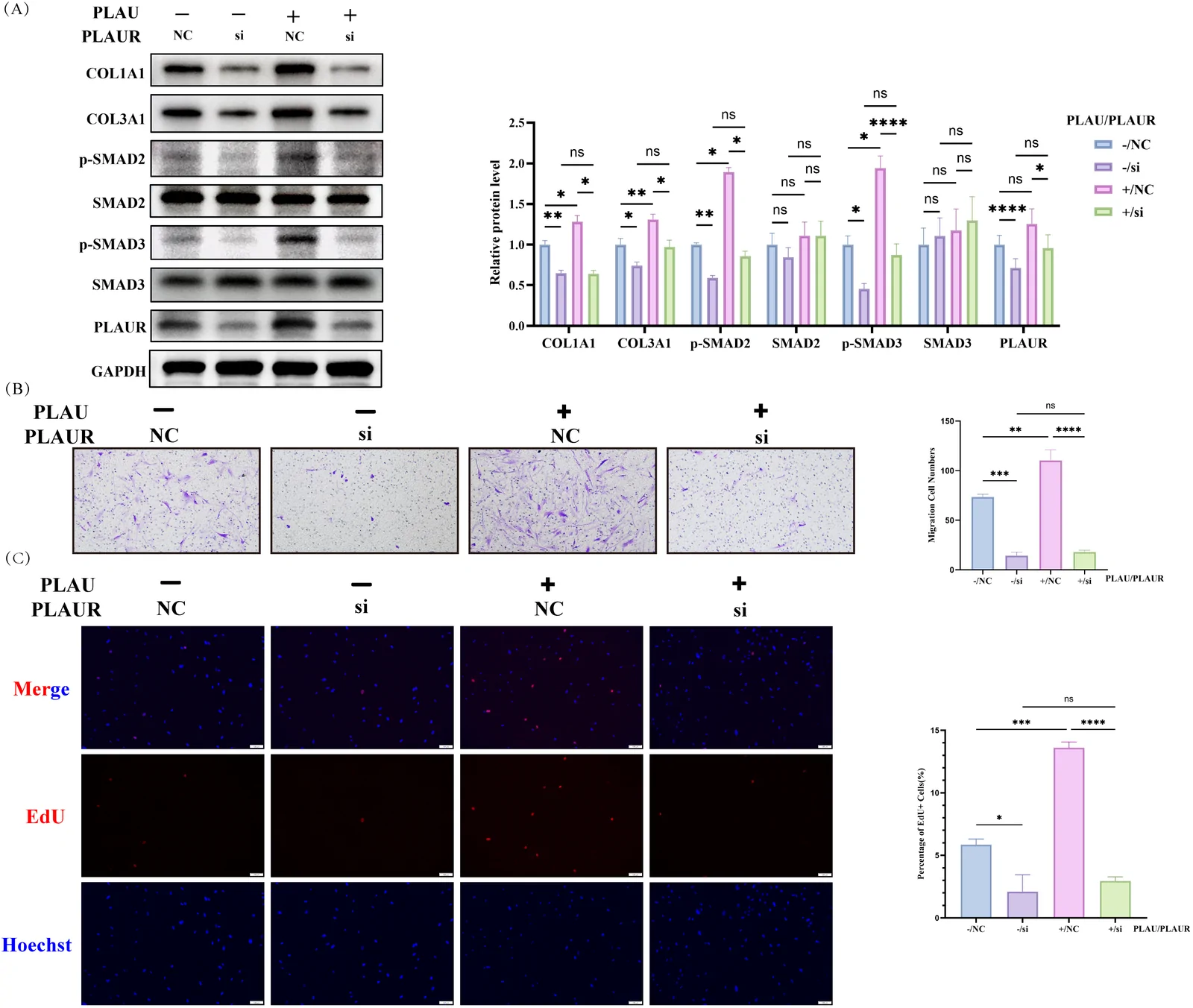
PLAU–induced TGF–β/Smad activation and fibroblast profibrotic responses are PLAUR–dependent. KFs were transfected with non–targeting control siRNA or PLAUR–targeting siRNA and subsequently stimulated with recombinant human PLAU protein. **(A)** Western blot analysis of COL1A1, COL3A1, p–SMAD2/3, SMAD2/3, and PLAUR under the indicated conditions, quantification of protein expression is shown on the right. **(B)** Representative images and quantitative analysis of Transwell assays showing fibroblast migration under the indicated conditions. **(C)** Representative fluorescence images and quantitative analysis of EdU incorporation assays showing fibroblast proliferation. EdU–positive cells are shown in red, and nuclei are counterstained with Hoechst (blue). These results demonstrate that PLAUR depletion attenuates PLAU–induced collagen expression, SMAD2/3 phosphorylation, migration, and proliferation. *P < 0.05, **P < 0.01, ****P < 0.0001; ns, not significant. Bar = 100 µm. PLAU, urokinase–type plasminogen activator; PLAUR, plasminogen activator urokinase receptor; COL1A1, collagen type I alpha 1 chain; COL3A1, collagen type III alpha 1 chain; p, phosphorylated; SMAD, suppressor of mothers against decapentaplegic; GAPDH, Glyceraldehyde–3–phosphate dehydrogenase; KFs, Keloid fibroblasts; EdU, 5–ethynyl–2′–deoxyuridine; NC, non–targeting control; si, small interfering RNA.

To investigate how fibroblasts respond to extracellular PLAU, we examined the involvement of its canonical receptor PLAUR/uPAR. KFs were transfected with PLAUR–targeting siRNA or non–targeting control siRNA and subsequently stimulated with recombinant PLAU protein. As shown in **Figure 7A**, PLAUR knockdown significantly reduced PLAUR expression. PLAU stimulation increased COL1A1 and COL3A1 expression and enhanced SMAD2/3 phosphorylation without affecting total SMAD2/3 levels, whereas these effects were markedly attenuated following PLAUR depletion. Furthermore, PLAUR knockdown reduced PLAU–induced fibroblast migration and proliferation (**Figures 7B, C**). These findings demonstrate that PLAU–induced fibroblast activation is PLAUR–dependent and involves activation of the TGF–β/Smad signaling pathway.

## 4 Discussion

This study integrates MR, scRNA–seq, and experimental validation to investigate the role of inflammatory mediators in keloid fibrosis. Through two–sample MR analysis of 91 inflammatory proteins, we identified five proteins (TNFRSF11B, EIF4EBP1, PLAU, IL–17A, and CCL25) with genetically supported associations with keloid susceptibility. Among these candidates, PLAU emerged as the most consistent candidate, showing increased expression in the keloid tissues across two independent scRNA–seq datasets and predominant enrichment in fibroblast populations. Subsequent functional studies demonstrated that PLAU promotes collagen expression, fibroblast proliferation and migration through PLAUR–dependent regulation of the TGF–β/Smad signaling pathway, positioning PLAU as an important mediator of keloid fibrogenesis.

Importantly, PLAU is not exclusively expressed in keloid fibroblasts. PLAU depletion in normal dermal fibroblasts had limited effects on basal collagen expression but reduced proliferation and migration, suggesting that PLAU also contributes to physiological fibroblast functions. However, elevated PLAU expression in keloid fibroblasts may amplify pathological profibrotic signaling, highlighting the need for therapeutic strategies that selectively target excessive PLAU activity while preserving normal tissue repair. Our PLAUR knockdown experiments support a PLAUR–dependent mechanism of PLAU–induced fibroblast activation. However, given the dual role of PLAUR in receptor signaling and plasminogen activation, plasmin–mediated mechanisms cannot be completely excluded.

The role of the urokinase plasminogen activator (uPA) system in fibrosis remains controversial, with previous studies reporting both profibrotic and antifibrotic effects. Our findings are consistent with observations by Zou et al., who reported increased expression of uPA, uPAR, and PAI–1 in fibrotic diseases, including keloids, hypertrophic scars, and scleroderma (16). Similarly, Vorstandlechner et al. demonstrated that PLAU knockdown and pharmacological inhibition of PLAU suppressed myofibroblast differentiation and extracellular matrix accumulation in fibroblast models (17). Together, these findings support the involvement of the PLAU/uPAR axis in fibrotic regulation and suggest PLAU as a potential therapeutic target.

Previous studies have provided mechanistic insights into the relationship between PLAU and TGF–β signaling. Briassouli et al. demonstrated that uPAR–dependent uPA activation can promote plasmin–mediated TGF–β activation and fibrosis (18). Similarly, Minami et al. showed that macrophage–derived uPA contributes to cardiac fibrosis through plasmin–dependent mechanisms involving profibrotic macrophage accumulation (19). In the present study, inhibition and rescue experiments demonstrated that PLAU regulates keloid fibroblast activation through the TGF–β/Smad pathway, supporting a functional connection between PLAU activity and TGF–β signaling in keloid fibrosis.

It is noted that previous studies have also reported antifibrotic effects of uPA in liver cirrhosis and pulmonary fibrosis models. For example, inducible lung–specific uPA expression reduced fibrosis after lung injury in mice (20). These apparently divergent effects may be explained by differences in tissue context, disease stage, and experimental conditions. We speculate that the profibrotic role of PLAU in keloids reflect the unique microenvironment of this chronic fibrotic disorder. Keloids represent a sustained, dysregulated wound healing response characterized by persistent inflammation and excessive ECM deposition. The keloid tissues examined in our study were obtained from relatively active disease stages, which may represent a pathophysiological state distinct from acute injury models or other fibrotic conditions. Another way to attribute the varying effects of PLAU in different studies would be by relating concentration–dependent and context–dependent effects. In our tests, human PLAU recombinant protein in small concentrations (0.2 and 2 ng/mL) significantly increased the production of collagen, growth, and migration of normal dermal fibroblasts. This finding suggests that physiological or modestly elevated PLAU levels in keloids may sustain TGF–β/Smad signaling through non–proteolytic mechanisms, such as modulating latent TGF–β activation or receptor–mediated signaling pathways, rather than through classical plasmin generation and ECM degradation. Indeed, Tuan et al. reported that although normal fibroblasts exhibited increased uPA and decreased PAI–1 level when cultured in collagen gels, there was no change in collagen accumulation, indicating that the net effect on fibrosis depends on the complex balance between uPA, PAI–1, and other regulatory components (21).

Our findings have potential clinical implications. MR analysis provides genetic evidence supporting an association between PLAU and keloid susceptibility, which is further supported by experimental validation. However, there are a few shortcomings that should be mentioned. First, MR analysis does not capture all aspects of PLAU regulation. In addition, MR analyses were based primarily on European ancestry GWAS datasets, whereas functional validation was performed in Chinese Han keloid samples. Although genetic associations may vary across populations due to differences in allele frequencies, linkage disequilibrium patterns, and genetic architectures, the consistent PLAU upregulation across independent scRNA–seq datasets and functional validation support the biological relevance of PLAU in keloid fibrosis. Future transethnic genetic studies are warranted to further validate these associations. Second, although scRNA–seq consistently identified PLAU enrichment in keloid fibroblasts, further characterization of fibroblast heterogeneity and temporal changes during keloid progression is needed. Third, recombinant PLAU stimulation may not fully recapitulate the complex *in vivo* microenvironment, where multiple cytokines, growth factors, and ECM components interact. Future studies should investigate PLAU dynamics during keloid formation and recurrence, evaluate potential combination therapies targeting PLAU and other profibrotic pathways, and explore local delivery strategies to maximize therapeutic efficacy while minimizing systemic effects.

## 5 Conclusions

In conclusion, integrated genetic, transcriptomic, and experimental evidence identifies PLAU as a genetically supported and biologically relevant mediator of keloid fibrosis. PLAU promotes fibroblast activation, including collagen production, proliferation, and migration, through PLAUR–dependent regulation of the TGF–β/Smad signaling pathway. These findings provide new insights into the inflammatory regulation of keloid pathogenesis and highlight PLAU as a potential therapeutic target for this challenging fibrotic disorder.

## Data Availability

The datasets analyzed during the current study are available in the GWAS Catalog repository (https://www.ebi.ac.uk/gwas/), Gene Expression Omnibus repository (GSE163973) and National Genomics Data Center, China National Center for Bioinformation (CNCB-NGDC) database (HRA000425). Moreover, the RNA-sequencing data generated in this study have been deposited in the CNCB-NGDC database under accession codes “HRA020520”.
