# Supplementary material for: PLAU promotes fibroblast activation and keloid fibrogenesis through PLAUR–Dependent TGF–β/Smad signaling
Source: Front Immunol. 2026 Sep 8;17:1855430. doi: 10.3389/fimmu.2026.1855430 (PMC13593517; doi:10.3389/fimmu.2026.1855430)
Supplement: Supplementary file 1 [file Supplementaryfile1.docx]

Supplementary Material

# 1 Supplementary Figures

**Supplementary Figure 1.** **Effects of PLAU knockdown in primary normal dermal fibroblasts. (A)** Western blot analysis of PLAU, COL1A1, and COL3A1 expression in primary normal dermal fibroblasts transfected with non-targeting control siRNA (NC) or two independent PLAU‒targeting siRNAs (si1 and si2), with corresponding quantitative analysis. **(B)** EdU incorporation assay assessing fibroblast proliferation following PLAU knockdown. **(C)** Transwell assay assessing fibroblast migration following PLAU knockdown. *p<0.05, **p<0.01; ns: not significant. Bar = 100 µm. PLAU: Urokinase‒type plasminogen activator; TGF‒β1: Transforming growth factor beta 1; NC: non‒targeting control; si: small interfering RNA (siRNA); COL1A1: Collagen type I alpha 1 chain; COL3A1: Collagen type III alpha 1 chain; GAPDH: Glyceraldehyde‒3‒phosphate dehydrogenase; EdU: 5‒ethynyl‒2’‒deoxyuridine.
